# Complication or Coincidence?

**Published:** 1894-02

**Authors:** J. W. Williams

**Affiliations:** Paterson, N. J.


### SIGNIFICANT NOTES.

The two following extracts from *The Medical News* will be amusing reading to homœopathists:

### COMPLICATION OR COINCIDENCE?

*To the Editor of The Medical News.*

Sir:—On the 8th of August, 1892, W. M., a young man, twenty-four years old, came to my office suffering from gonorrhœa of moderate severity.

Under treatment with daily irrigations of the urethra, with warm, mild antiseptic solutions and appropriate internal medication, the urethritis almost disappeared, with no complication, at the end of two weeks. Then the patient complained of muscular pain in the right arm and shoulder, so severe as to render him unable to use the arm for any purpose. Examination disclosed spots of tenderness over the upper third of the biceps and middle of the anterior half of the deltoid. There was slight swelling and heat about the joint, but no stiffness or crepitus, and no pain on manipulation. There was, however, marked pain on active motion.

The temperature with the onset of the pain rose to 103° F. At the end of the first day this was reduced to 100° F. by the use of large doses of Quinine.

The man was given 10-grain doses of Sodium Salicylate three times daily. The shoulder was blistered by an application of Cantharidal Collodium. In four days the pain had disappeared, but the patient discovered that the power of abducting the arm was completely lost.

Examination showed complete paralysis of the deltoid, with some tenderness over its lower half.

There was no stiffness, or crepitus, or pain in the joint.

The patient was put on Potassium Iodide in 10-grain doses. There has been no improvement in the condition at the end of a week.

There was no history of traumatism.

Could the condition be due to gonorrhœal infection, or was it merely a coincidence?

Respectfully,

J. W. Williams, A. M., M. D.

Paterson, N. J.

—*Philadelphia Medical News, Saturday, Sept. 24th*, 1892.

### BEE-VIRUS FOR ACUTE RHEUMATISM.

*To the Editor of The Medical News.*

